# Knowledge of Statistics or Statistical Learning? Readers Prioritize the Statistics of their Native Language Over the Learning of Local Regularities

**DOI:** 10.5334/joc.209

**Published:** 2022-02-21

**Authors:** Jarosław R. Lelonkiewicz, Michael T. Ullman, Davide Crepaldi

**Affiliations:** 1 Scuola Internazionale Superiore di Studi Avanzati (SISSA), Trieste, Italy; 2 Georgetown University, Washington D.C., USA

**Keywords:** statistical learning, reading, visual word processing

## Abstract

A large body of evidence suggests that people spontaneously and implicitly learn about regularities present in the visual input. Although theorized as critical for reading, this ability has been demonstrated mostly with pseudo-fonts or highly atypical artificial words. We tested whether local statistical regularities are extracted from materials that more closely resemble one’s native language. In two experiments, Italian speakers saw a set of letter strings modelled on the Italian lexicon and guessed which of these strings were words in a fictitious language and which were foils. Unknown to participants, words could be distinguished from foils based on their average bigram frequency. Surprisingly, in both experiments, we found no evidence that participants relied on this regularity. Instead, lexical decisions were guided by minimal bigram frequency, a cue rooted in participants’ native language. We discuss the implications of these findings for accounts of statistical learning and visual word processing.

One of the most striking features of the human mind is its ability to rapidly learn about regularities that are present in the environment. In a matter of minutes, people can implicitly detect and acquire a range of information patterns, including distributional variability, frequency, and probability with which events co-occur in the perceptual stream. Impressively, this type of learning, often referred to as *statistical learning*, has been observed across different tasks, contexts, and cognitive domains (for reviews, see Aslin, 2017; Aslin & Newport, 2012; Christiansen, 2019; Frost, Armstrong, & Christiansen, 2020; Newport, 2016; Perruchet & Pacton, 2006; Saffran & Kirkham, 2018; Thiessen, Kronstein, & Hufnagle, 2013).

One domain where statistical learning has been particularly well-evidenced is visual processing. In a landmark study by Fiser and Aslin (2001), adult participants were exposed to a stream of visual scenes composed of multiple abstract shapes. They were given no instructions beyond being asked to attend to the stimuli. Unknown to the participants, some shapes were repeatedly presented next to one another, thus constituting frequent pairs. Next, in a two-alternative forced choice test, participants were asked to judge the familiarity of shape pairs, on each trial choosing between a frequent pair and a pair that had never occurred in the scenes. The results revealed that frequent pairs were more often judged as familiar, suggesting that participants had implicitly learnt the frequency with which pairs occurred across the scenes (see also Fiser & Aslin, 2002; 2005; Turk-Browne, Jungé, & Scholl, 2005). Further research extended these findings by showing that statistical learning is a feature of visual processing already in infancy (Bulf, Johnson, & Valenza, 2011; Slone & Johnson, 2018), and that it occurs for different types of visual stimuli, including complex object arrays (Orbán, Fiser, Aslin, & Lengyel, 2008), real-world scenes (Brady & Oliva, 2008), and faces (Turk-Browne, Scholl, Johnson, & Chun, 2010).

Given its prevalence in the visual domain, several theoretical accounts have claimed that statistical learning is implicated in the processing of written language. For instance, Frost (2012) observed that languages provide readers with a rich array of probabilistic regularities and argued that efficient reading relies on the fact that cognitive system is tuned to pick up this information (e.g., in Semitic languages word roots are formed using only a small number of letters, and so focusing on these letters could aid in word identification). Similarly, Grainger and Ziegler (2011) suggested that reading involves the extraction of letter co-occurrence statistics: When processing words along a fine-grained route (one of the two modes of orthographic processing proposed by Grainger and Ziegler 2011), readers identify the frequently co-occurring letters and chunk them into higher–level orthographic representations (e.g., morphemes). Numerous other accounts have converged on the idea that the ability to extract regularities from the written input is important for reading, as it supports the acquisition of orthographic codes (Dehaene, Cohen, Sigman, & Vinckier, 2005; Grainger, Dufau, & Ziegler, 2016), lexical representations (Frost, Kugler, Deutsch, & Forster, 2005), and reading skills at large (Arciuli, 2018; Arciuli & Simpson, 2012; Seidenberg & MacDonald, 2018).

But do people actually pick up on visual statistical cues as they read? Interestingly, there is some evidence that this might be the case. The ability to extract visual regularities appears to be present in several animal species, including primates. In Grainger et al. (2012), baboons saw foil strings designed to imitate English words intermixed with actual words, and received a reward every time they correctly identified a word. Since they could not rely on linguistic knowledge (e.g., semantics or phonology), the animals were given a visual cue: foil strings had lower average bigram frequency (ABF) than words, meaning that – on average – they were composed of letter bigrams that occurred less frequently within the stimuli set. The study found that baboons were able to correctly distinguish between foils and words even for items they had not seen before (and thus could not have remembered), implying that they had picked up on the purely visual information about bigram frequency (similar findings have been reported for chicks and pigeons; see Santolin & Saffran, 2018). However, though suggestive, evidence from animal studies cannot ultimately tell whether human readers extract statistics from written words.

Two recent artificial orthography studies brought us closer to answering this question: In Chetail (2017), adult readers were exposed to a large (e.g., 320 items in Experiment 1a) lexicon of strings composed of unfamiliar psuedofonts. Importantly, the stimuli involved some highly frequent bigrams. As in the classic visual statistical learning experiments (e.g., Fiser & Aslin, 2001), participants were asked to observe the stimuli with no mention of any implicit regularities. And yet, they acquired the bigram statistics – in a subsequent wordlikeness task, previously unseen strings were judged as more word-like if they involved frequent bigrams. Using a similar paradigm, Lelonkiewicz, Ktori, and Crepaldi (2020) exposed readers to 200 pseudofont strings that contained chunks of frequently co-occurring characters. Subsequently, string processing came to resemble that of real morphologically complex words (i.e., readers became sensitive to the presence and position of chunks within strings, a finding previously observed for morphological affixes within words; see Crepaldi, Rastle, & Davis, 2010; Crepaldi, Rastle, Davis & Lupker, 2013), implying that participants had learnt the character co-occurrence statistics and used this information to decompose the pseudofont strings into chunks. While allowing good experimental control (e.g., ruling out the confounds related to semantic representations), these experiments have the obvious caveat of involving stimuli made of unfamiliar, non-linguistic symbols, which makes it difficult to understand to what extent they apply to real language processing.

To partially circumvent this issue, several studies investigated visual statistical learning in nonwords composed of familiar letters and generated using artificial grammars. For example, Ise et al. (2012) asked children to memorize and pronounce 15 such nonwords; next, children saw further nonwords and decided if these new items belonged to the same fictitious language as the memorized ones. The results were consistent with statistical learning – nonwords that contained bigrams or trigrams that frequently occurred within the memorized set were more often ascribed to the fictitious language. In a similar vein, Samara and Caravolas (2017) showed that adult readers were more likely to judge novel letter strings as grammatical if they were composed of chunks that were frequent within a set of 24 previously memorized nonwords (see also Nigro, Jiménez-Fernández, Simpson, & Defior, 2015; 2016; Maurer et al., 2010; Taylor, Davis, & Rastle, 2017).

These artificial grammar experiments go in line with the claim that people discover local statistical regularities as they read. However, their conclusions are based on a very particular case of language processing: Readers were first exposed to small and highly artificial sets of learning items and then engaged in a visual word processing task in which they received no information about the accuracy of their performance; in contrast, language learning likely benefits from some form of feedback (Dale & Christiansen 2004; Pashler, Cepeda, Wixted, & Rohrer, 2005; Rastle, Lally, Davis, & Taylor, 2021).

In the present study, we set out to extend the current evidence. To this end, we devised a paradigm capitalising on the strengths of the previous designs and addressing some of their weaknesses. In two experiments, we examined if human readers extract statistical regularities while processing a large lexicon of strings (400 distinct items). To ensure a more naturalistic context (as compared to baboons learning English words or humans reading an unfamiliar alphabet), we modelled the stimuli on real language (participants’ native Italian) and used familiar letters. (See Discussion for how this relates to real-world reading contexts, such as learning to read new words in one’s native language or learning to read in a new language.) However, to minimise the chance that the processing would be guided by the information associated with the lexical level (e.g., word meaning, word frequency), we used strings that were all nonwords. We hypothesised that, if the ability to extract regularities plays a role during visual word processing, readers should pick up on the statistical characteristics of the new lexicon.

To test this hypothesis, in Experiment 1 we presented Italian speakers with a set of letter strings and asked them to guess which strings were words in a fictitious language and which were not (henceforth, *words* and *foils*). Importantly, and unbeknownst to participants, fictitious words had higher average bigram frequency (ABF) than foil strings. Much like in Grainger et al. (2012), participants made their guesses after every string they saw and we expected them to implicitly learn the ABF pattern as they progressed with the experiment. To foster learning, each string was shown three times and feedback was given after each guess. We hypothesised that if participants are indeed sensitive to statistical characteristics of learning materials, they should pick up on the ABF pattern underlying the words vs. foils distinction and use it in their guesses. In Experiment 2, we further increased the ABF difference between words and foils and once again tested if participants would use this cue.

Further, to help understand which cognitive faculties may impact the learning of local regularities in reading, we collected additional measures of statistical learning in a non-verbal visual task (Experiment 1); of learning in declarative and procedural memory (Experiment 2); and of vocabulary span (Experiments 1–2). Statistical learning has been hypothesized to tie to a range of individual abilities (e.g., Siegelman & Frost, 2015; Arciuli, 2017; Schmalz, Altoè, & Mulatti, 2017). We reasoned that a positive correlation between the use of ABF and visual statistical learning would provide further evidence that participants acquired this cue during the task. Conversely, a positive correlation with participants’ vocabulary span would link the ability to use the cue to pre-existing linguistic knowledge.

The declarative and procedural memory measures aimed to identify possible neurocognitive substrates of regularity learning in reading. Specifically, to understand whether any such learning was linked to declarative memory (defined as the learning and memory that is rooted in the hippocampus and associated circuitry) and/or procedural memory (the learning and memory rooted in the basal ganglia and associated circuitry) (Ullman, Earle, Walenski, & Janacsek, 2020), we tested for positive correlations between the use of ABF and either memory measure. Notably, a correlation with procedural memory would strengthen the hypothesis that participants acquire regularities through implicit learning, since procedural memory seems to underlie only the acquisition of implicit knowledge (Ullman et al., 2020). In contrast, a correlation with declarative memory would be consistent with participants having learned the statistic explicitly, since this system is critical for explicit knowledge.

## Experiment 1

### Methods

The stimuli and experimental scripts used in the main task, as well as raw data and commented analysis scripts can be found at the project’s Open Science Framework page: *https://osf.io/vc6rw/.*

#### Participants

We recruited 45 monolingual native Italian speakers who did not use any other languages on a daily basis, had normal or corrected-to-normal vision, no learning or language disorders, and were 18–35 years old. All participants gave informed consent prior to the experiment and received 18€. All experimental procedures were approved by the Ethics Committee at Scuola Internazionale di Studi Superiori Avanzati (SISSA), where participants were tested.

#### Apparatus, stimuli and procedure

Participants carried the experiment out individually, in a sound-proof booth. Prior to the main task (lexical judgment), participants completed a measure of vocabulary span (MHVS) and a visual statistical learning task (VSL). All instructions were given in Italian.

##### Mill-Hill Vocabulary Scale (MHVS)

First, participants completed a paper-based scale where they saw 28 Italian words and for each word selected the correct synonym from a list of six alternatives (we used an Italian translation of the MHVS originally introduced as a companion to Raven’s Progressive Matrices; Raven, 1958). Vocabulary span was operationalized as the number of correct responses out of the total of 28, meaning that high numbers were indicative of a broad vocabulary. The scale took 5–10 minutes to complete.

##### Visual Statistical Learning (VSL)

Next, participants sat in front of a computer and performed a variant of a commonly used VSL task (e.g., Frost, Siegelman, Narkiss, & Afek, 2013; Turke-Brown et al., 2005). The task started with a familiarization phase, where participants saw 16 abstract shapes appearing one-by-one in a continuous stream (800 ms ON, 200 ms OFF). Unbeknownst to participants, shapes were arranged in 8 triplets, each appearing 24 times (triplets appeared in a pseudo-random order with the constraint that the same triplet cannot be immediately repeated). Thus, shapes within a triplet had much higher transitional probabilities (TPs = 1) than shapes between triplets (TPs = 1/7) or shapes that never appeared one after another (TPs = 0). In the testing phase, we investigated if participants successfully learnt these probabilities: Participants were presented with 34 trials where they chose between sequences composed of shapes with high TPs and “foil” sequences composed of shapes with low TPs (16 trials were 2AFC triplets, 6 trials were 2AFC pairs, 4 trials were 4AFC pairs, and 8 trials were 4AFC triplets; trials were presented in random order), and further 8 trials where they saw an incomplete sequence and they selected the shape that they thought was missing (4 trials involved a pair, and 4 trials a triplet). We operationalized visual statistical learning ability as the number of correct responses out of the total of 42, so that the higher the number, the better the individual’s ability to learn visual regularities. Participants sat in front of a 27’’ BenQ XL2720 monitor and responded using an English QWERTY keyboard. The experiment was delivered with Presentation (v19.0, Neurobehavioral Systems, Inc., Berkeley, CA, *www.neurobs.com*). Participants completed the VSL task in about 15 minutes.

##### Lexical Judgment

Participants then moved onto the primary task of the study: They were told they would see words in a fictitious “Turistino” language, as well as combinations of letters that were similar, but were “something else”. The task was to distinguish between strings that were fictitious words (e.g., *etesse*) and foil strings (e.g., *spizio*). Participants were given no further information on how to tell these two types apart.

The stimuli were all nonwords, but to increase the chance that they would be processed similarly to real words, we ensured that they were pronounceable and consisted of bigrams and letters naturally occurring in participants’ native Italian. Word strings and foils were similar to one another: They were comparable in length (either were 4–6 letters long; Mdn_words_ = 6, Mdn_foils_ = 6; *U* = 20113, *p* = .907; non-parametric Man-Whitney U test, two-sided) and constructed using identical sets of 150 unique bigrams and 21 unique letters. They were also orthographically equidistant to Italian (as captured by Coltheart’s N; Mdn_words_ = 0, Mdn_foils_ = 0; *U* = 20770; *p* = .119).

**Figure 1:**
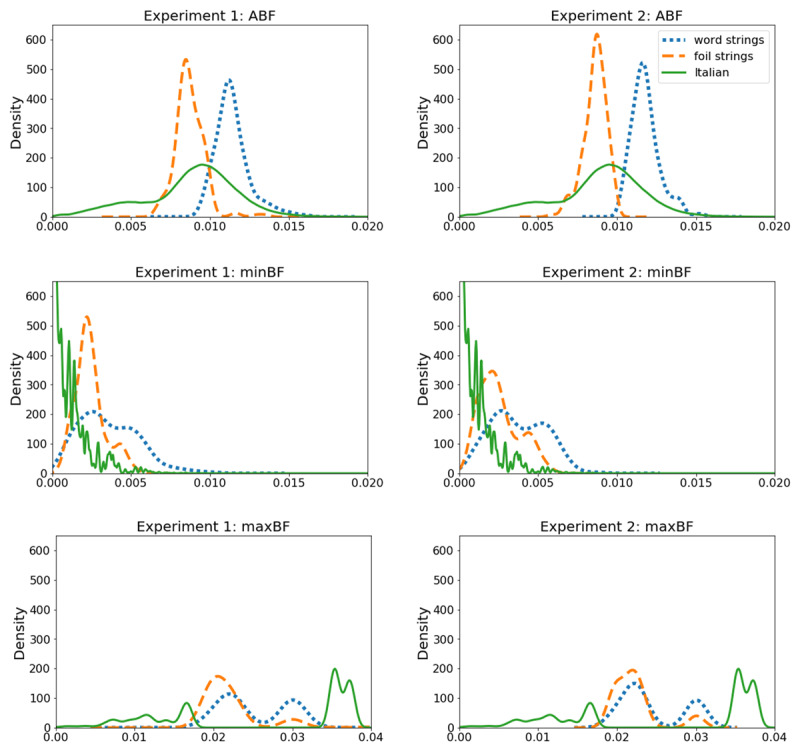
Experiments 1–2: Density plots illustrating average bigram frequency (ABF), minimal bigram frequency (minBF), and maximal bigram frequency (maxBF) for fictitious words, foils, and real words in the Italian lexicon.

**Figure 2:**
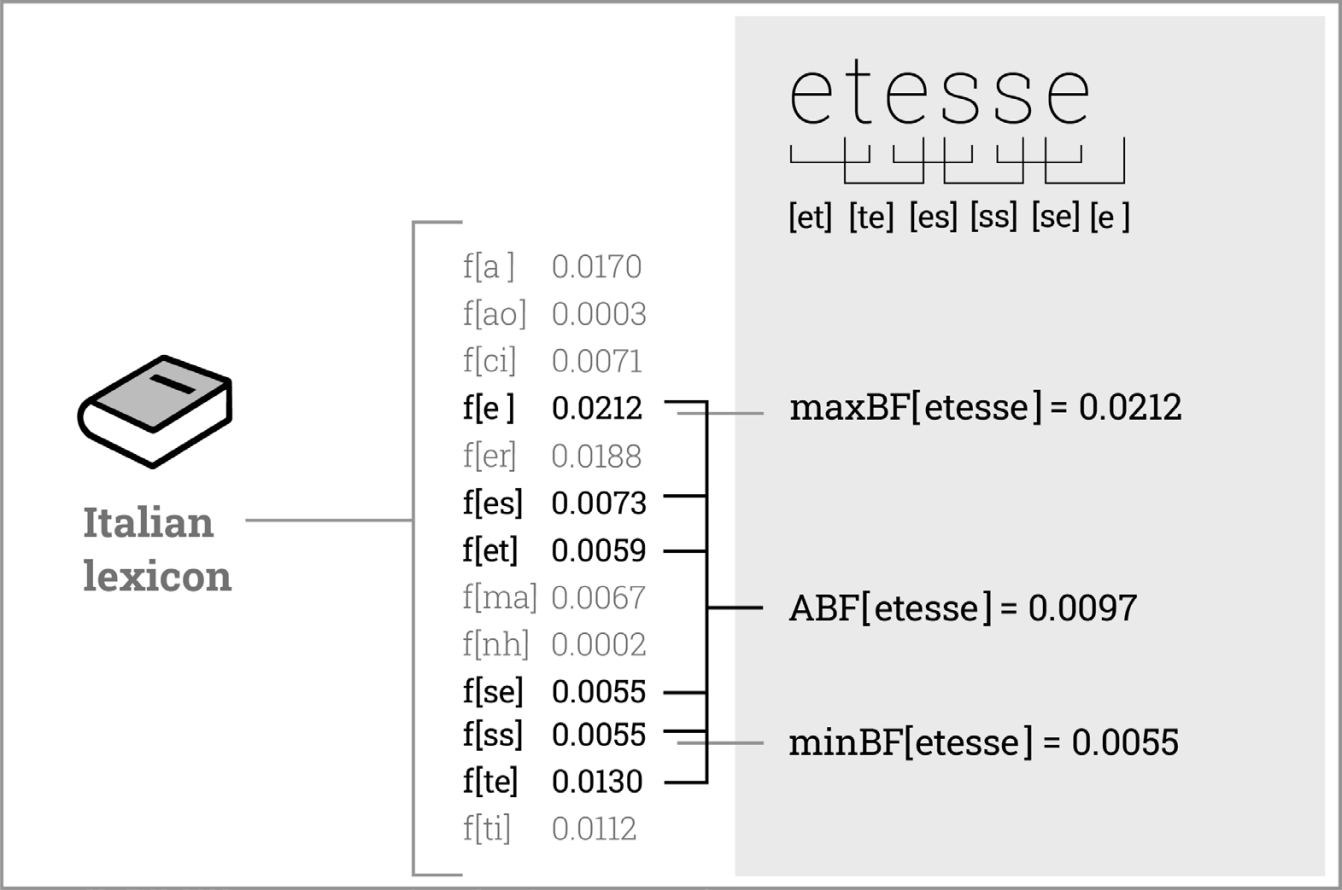
Experiments 1–2: An illustration of how average bigram frequency (ABF), minimal bigram frequency (minBF), and maximal bigram frequency (maxBF) were calculated. For each of the novel strings (e.g., *etesse*), we identified all bigrams present in this string and matched them with the frequencies with which these bigrams occur in the Italian lexicon (SUBTLEX-IT; Crepaldi et al., 2015; Italian bigram frequencies were calculated as position-independent occurrence probabilities). ABF was defined as the average value of the frequencies of all the bigrams present in that string, minBF was equivalent to the frequency of the bigram with the lowest frequency from all the bigrams present in this string (e.g., f[ss] = 0.0055), and maxBF was equivalent to the frequency of the bigram with the highest frequency (e.g., f[e ] = 0.0212).

Critically, however, word strings differed from foils insofar that they tended to have higher average bigram frequency (ABF; see ***Figure 1***). ABF was defined as the average frequency of all closed bigrams present in a string. Frequencies of individual closed bigrams were based on the Italian lexicon (SUBTLEX-IT; Crepaldi et al., 2015). They were calculated as occurrence probabilities (i.e., count of the occurrences of a given bigram in the Italian lexicon divided by the count of all occurrences of all bigrams in this lexicon), in a position-independent manner (i.e., all occurrences of a given bigram were counted towards a single score, regardless of the bigram position within words). See ***Figure 2*** for an illustration of how ABF was calculated.

Participants were given no explicit information about the ABF pattern, and were expected to learn it on their own as they were doing the task. To support such learning, each lexical judgment was followed by a feedback message telling the accuracy of the judgment and revealing whether the string was a fictitious word or a foil. However, we did not know a priori if participants would pick up on the ABF pattern underlying the word vs. foil distinction. Specifically, given the high degree of similarity between the experimental strings and real Italian words, it was plausible that participants, all native speakers of Italian, could instead rely on some other regularities derived from the Italian lexicon. For example, lexical judgments could be guided by the presence of bigrams that are particularly rare or particularly common in Italian. To capture this possibility, for each string we calculated minimal bigram frequency (minBF), defined as the frequency of the bigram with the lowest Italian-based frequency from all the bigrams present in this string, as well as maximal bigram frequency (maxBF), defined as the frequency of the bigram with the highest Italian-based frequency (for an illustration, see ***Figure 2***). This allowed us to control for a possible effect of these metrics at the stage of statistical analysis.

A noteworthy aspect of our method is that the bigram frequencies used in the calculation of ABF, minBF, maxBF were based on the Italian lexicon. However, another approach could be to calculate bigram frequencies based on how often the bigrams occurred within the experimental stimuli. After all, participants could use such local frequencies to distinguish between word strings and foils, of course providing there would be a systematic difference between the two types (e.g., a certain bigram could be very frequent in words, but not in foils). Similarly, participants could rely on local letter frequencies.

***Table 1*** offers a comparison between the frequency statistics as calculated based on Italian lexicon vs. on the experimental stimuli. As illustrated in ***Table 1***, whereas word strings were similar to foils with regards to letter frequency statistics, they differed in terms of bigram frequencies. This was most likely due to the nature of our manipulation (i.e., word strings were designed to have higher average bigram frequency than foils, which might have led to some bigrams being used more often in words than foils). We return to this point in the Results section (see the passages reporting the interaction between Type and Seen).

**Table 1:** Experiments 1–2: A correlation matrix (Spearman’s ρ; all *p*s < .01) illustrating the similarity in letter and bigram probabilities as calculated based on fictitious word strings, foils, and the Italian lexicon.


EXPERIMENT 1				
		ITALIAN	WORDS	FOILS

letter probabilities	Italian	–	0.97	0.91

	words	0.97	–	0.92

	foils	0.91	0.92	–

bigram probabilities	Italian	–	0.78	0.43

	words	0.78	–	0.37

	foils	0.43	0.37	–

**EXPERIMENT 2**				

		**ITALIAN**	**WORDS**	**FOILS**

letter probabilities	Italian	–	0.96	0.86

	words	0.96	–	0.86

	foils	0.86	0.86	–

bigram probabilities	Italian	–	0.84	0.35

	words	0.84	–	0.28

	foils	0.35	0.28	–


Returning to the description of the lexical judgment task, in each trial participants saw a fixation cross (500 ms), followed by either a word string or a foil, appearing centrally on the screen. After participants’ response (or after 2000 ms has lapsed, whichever occurred first), the string would be replaced by a feedback message (“*Corretto/Scorretto! Questa era una parola/nonparola*”; English translation:“*Correct/Incorrect! This was a word/nonword*”). Participants completed 1200 trials organised into 10 blocks: There were 200 words and 200 foils, each stimulus shown three times, presented in random order, with the constraint that all repetitions of a stimulus had to occur within the same block.

Stimuli were displayed in black, against a light gray background (#f0f0f0). Participants operated the same set-up as in the VSL task, except here they responded by pressing F/J (response type to key assignment was counter-balanced between participants). The experiment was programmed in OpenSesame (v3.1.9; Mathôt, Schreij, & Theeuwes, 2012). The lexical judgment task took about 60 minutes to complete.

### Statistical Analysis

Prior to analysis, we removed 2 participants whose data was not usable (1 due to data corruption, 1 due to a technical failure; leaving n = 43 in the analysis).

Based on the hypothesis that readers can discover the implicit statistical structure of learning materials, we predicted that lexical judgments in our task would be guided by ABF. Specifically, we expected that the likelihood of judging a string to be a fictitious word should be positively related to this string’s ABF.

To test this prediction, we conducted a binomial generalized linear mixed model (GLMM) estimating the odds of making a “word” response. We included ABF, Type (foil vs. word), and Seen (unseen vs. seen; i.e., whether at the time of making the response participant had already seen the string) as predictors, as well as random by-participant intercepts and slopes for the three-way interaction between ABF, Type, and Seen, and by-item intercepts and slopes for Seen. We applied effect contrast-coding to the factorial predictors (Type, Seen), and scaled and centred the continuous predictor (ABF). The model had the following structure: “word” response ~ (ABF * Type * Seen) + (ABF * Type * Seen | Participant) + (Seen | Item). All GLMM’s reported in this paper were computed using *lme4* R package (*lme4* version 1.1–14; Bates, Maechler, Bolker, & Walker, 2015; R version 3.4.2; R Core Team, 2017).

Moreover, we wanted to test for the possibility that lexical judgments would be driven by a different measure of bigram frequency, one that was not reinforced during the lexical judgment task, but could nevertheless influence participants’ performance. Thus, we ran two further models based on the GLMM described above: One including minBF as an additional predictor, and another including maxBF. As before, we contrasted the factorial predictors and scaled and centred the continuous ones. The models had the following structure: “word” response ~ (ABF * Type * Seen) + (minBF/maxBF * Type * Seen) + (ABF * Type * Seen | Participant) + (minBF/maxBF * Type * Seen | Participant) + (Seen | Item).

To determine which model provided the best fit to our data, we compared the three models in terms of explained variance. We found that the ABF + minBF model was superior to the other two (i.e., the ABF model, and the ABF + maxBF model; *p*s < .001; comparisons were done using the *anova* function; base R). For this reason, throughout this paper, we consider this model to provide the primary representation of our data (results from the other two models can nevertheless be found in the Supplement; see Tables S15 and S16).

Apart from investigating our main hypothesis, we wanted to explore whether the effects of statistical cues on lexical judgment would increase over time, thus indicating that participants were learning to rely on such cues as they progressed through the task (prior to this analysis we excluded 1 further participant who did not produce responses in the last two blocks of the experiment; leaving n = 42). To this end, we re-fit the ABF + minBF model including also experimental block as a predictor (scaled): “word” response ~ (ABF * Type * Seen * Block) + (minBF * Type * Seen * Block) + (ABF * Type * Seen * Block | Participant) + (minBF * Type * Seen * Block | Participant) + (Seen | Item).

Finally, to investigate if the use of statistical cues was related to participants’ vocabulary span or their ability to learn visual regularities, we re-fit the ABF + minBF model including participants’ MHVS score, and (as a second, separate model) re-fit it including participants’ VSL score (MHVS/VSL scores were scaled and centred). The models followed the structure: “word” response ~ (ABF * Type * Seen * MHVS/VSL) + (minBF * Type * Seen * MHVS/VSL) + (ABF * Type * Seen * Block | Participant) + (minBF * Type * Seen * Block | Participant) + (Seen | Item).

### Results

**Figure 3:**
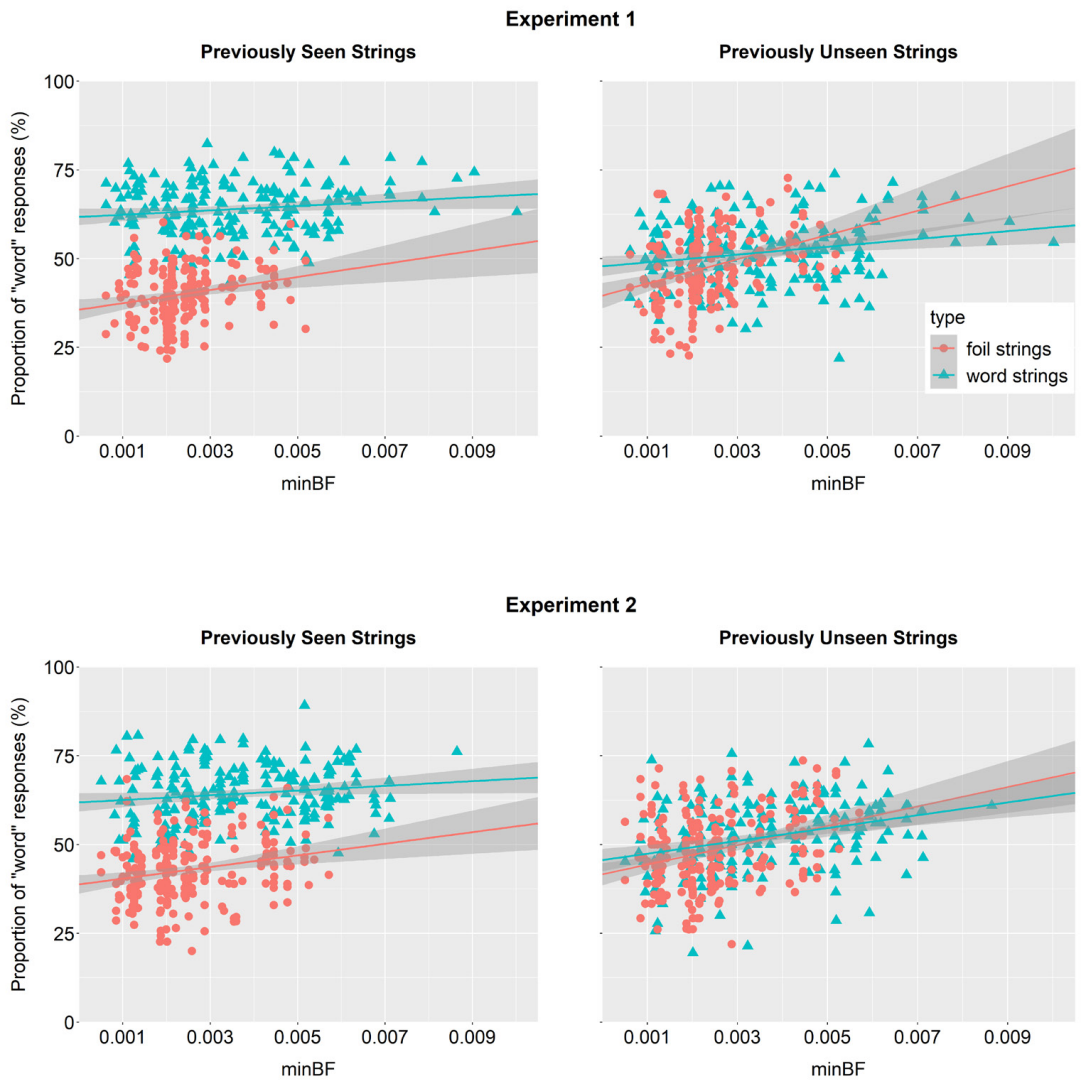
Experiments 1–2: Mean percentages of “word” responses for each item, shown by Type (foil vs. word strings) and Seen (previously unseen vs. seen). The fitted lines represent the effect of minimal bigram frequency (minBF).

The main findings are visualised in ***Figure 3*** and model summaries are reported in ***Table 2***. First, we observed a main effect of Seen (*p* < .001), which was further qualified by a significant interaction with Type (*p* < .001), indicating that although the odds of making a “word” response were overall higher for word strings than foils, this effect was greater in strings that participants had already seen compared to previously unseen strings. Moreover, a follow-up analysis revealed that the effect of Type was significant for previously seen items (*p* < .001), but not for unseen items (*p* = .651; full results in Table S1), meaning that participants were able to successfully distinguish between fictitious words and foils only when judging items for which they had already received feedback (recall that after responding to a string participants received feedback that revealed the identity of a string). Conversely, when judging items on which they had not received feedback, they could not tell the two types apart (***Figure 3***; see also Figure S1 in the Supplement showing the proportion of “word” responses by the first, second, and third presentation). An important implication of this finding is that it suggests that participants did not base their judgments on the differences in the statistical characteristics of word strings vs. foils (if there would be any systematic difference between words and foils that the participants could use, we would expect them to successfully distinguish between words and foils also for previously unseen strings).

**Table 2:** Experiments 1–2: Results from the fixed effects structure of the GLMM’s including ABF, minBF, Type (foil vs. word) and Seen (unseen vs. seen).


EXPERIMENT 1				

	\hat \beta	*SE*	*z*	*p*

Intercept	.06	.09	.67	.504

ABF	.06	.07	.92	.356

Type	–.04	.08	–.50	.620

Seen	–.32	.07	–4.85	**<.001**

minBF	.22	.06	3.62	**<.001**

ABF * Type	.00	.08	.02	.981

ABF * Seen	.07	.06	1.10	.273

Type * Seen	.87	.11	7.98	**<.001**

minBF * Type	–.16	.07	–2.35	**.018**

minBF * Seen	–.11	.05	–2.00	**.045**

ABF * Type * Seen	–.11	.08	–1.47	.142

minBF * Type * Seen	.09	.06	1.49	.137

**EXPERIMENT 2**				

	\hat \beta	***SE***	***z***	***p***

Intercept	.06	.09	.71	.473

ABF	.08	.07	1.07	.285

Type	–.10	.09	–1.15	.252

Seen	–.26	.08	–3.05	**.002**

minBF	.14	.04	3.46	**<.001**

ABF * Type	.02	.10	.25	.800

ABF * Seen	–.01	.07	–.15	.877

Type * Seen	.71	.13	5.55	**<.001**

minBF * Type	–.04	.05	–.93	.355

minBF * Seen	–.05	.04	–1.15	.250

ABF * Type * Seen	.12	.09	1.31	.190

minBF * Type * Seen	–.02	.05	–.52	.603


In line with this finding, the analysis also revealed no effects of ABF (*p*s > .141), implying that participants did not use this cue for lexical judgments. Instead, they relied on minBF: We observed a significant main effect of minBF, such that the higher the string’s minBF, the greater the odds of judging this string to be a fictitious word (*p* < .001). Further, this effect was qualified by a significant interaction with Seen (*p* = .045) and by a separate interaction with Type (*p* = .018), suggesting that minBF had a particularly strong impact when participants were responding to previously seen strings or to foils (see ***Figure 3***; these findings were confirmed in a model controlling for string length, and another model controlling for orthographic similarity; see Tables S11–14).

The unexpected finding that lexical judgments were guided by minBF prompted us to ask if participants were somehow able to learn to use this cue during the task. However, the model controlling for the effect of experimental block found no evidence for such learning: Whereas the effect of minBF was once again present (*p* = .0498), there was no main effect of Block and no significant interactions including this predictor (*p*s > .082), suggesting that the tendency to rely on minBF was unlikely acquired during the experiment. Further, there were no interactions involving ABF and Block (*p*s > .679), implying that, despite receiving feedback, participants did not learn to use ABF over the course of the experiment (full results in ***Table 3***).

**Table 3:** Experiments 1–2: Results from the fixed effects structure of the GLMM’s including ABF, minBF, Type (foil vs. word), Seen (unseen vs. seen), and controlling for Block.


EXPERIMENT 1				

	\hat \beta	*SE*	*z*	*p*

Intercept	.06	.12	.47	.636

Block	–.01	.11	–.12	.906

ABF	.07	.10	.76	.448

Type	–.24	.13	–1.86	.062

Seen	–.33	.13	–2.44	**.015**

minBF	.18	.09	1.96	**.0498**

ABF * Block	–.02	.10	–.20	.838

Type * Block	.22	.13	1.73	.083

ABF * Type	.07	.15	.51	.607

Seen * Block	.00	.13	.00	.997

ABF * Seen	.07	.13	.52	.603

Type * Seen	1.05	.18	5.63	**<.001**

minBF * Block	.03	.10	.28	.778

minBF * Type	–.15	.11	–1.34	.181

minBF * Seen	.02	.12	.14	.891

ABF * Type * Block	–.06	.14	–.41	.680

ABF * Seen * Block	.00	.12	.02	.986

Type * Seen * Block	–.19	.17	–1.07	.285

ABF * Type * Seen	–.15	.18	–.79	.428

minBF * Type * Block	.00	.11	–.04	.967

minBF * Seen * Block	–.12	.12	–1.02	.306

minBF * Type * Seen	–.01	.14	–.09	.925

ABF * Type * Seen * Block	.03	.18	.19	.850

minBF * Type * Seen * Block	.10	.13	.77	.443

**EXPERIMENT 2**				

	\hat \beta	***SE***	***z***	***p***

Intercept	–.09	.14	–.68	.494

Block	.17	.13	–1.26	.207

ABF	–.04	.14	–.34	.736

Type	–.06	.17	–.38	.704

Seen	–.20	.17	–1.17	.242

minBF	.26	.07	3.49	**<.001**

ABF * Block	.14	.13	1.03	.303

Type * Block	–.05	.17	–.31	.754

ABF * Type	.19	.17	1.09	.274

Seen * Block	–.08	.15	–.55	.583

ABF * Seen	.11	.16	.68	.497

Type * Seen	.75	.22	3.35	**<.001**

minBF * Block	–.13	.08	–1.71	.087

minBF * Type	–.14	.09	–1.62	.105

minBF * Seen	–.19	.10	–1.92	.055

ABF * Type * Block	–.16	.17	–.97	.332

ABF * Seen * Block	–.13	.16	–.85	.398

Type * Seen * Block	–.02	.20	–.10	.922

ABF * Type * Seen	–.01	.22	–.06	.949

minBF * Type * Block	.12	.09	1.25	.211

minBF * Seen * Block	.15	.09	1.63	.103

minBF * Type * Seen	.11	.11	.99	.323

ABF * Type * Seen * Block	.15	.21	.72	.468

minBF * Type * Seen * Block	–.16	.11	–1.43	.152


Finally, with regards to our secondary measures, we found that the effect of minBF was positively related to participants’ vocabulary span (*p* = .008), such that the higher the individual’s MHVS score, the stronger the effect of minBF; there were no other reliable effects involving this measure (*p*s > .050; full results in Table S3). Participants’ ability to learn visual regularities as measured by the VSL task had no effect on performance in our task (*p*s > .208; full results in Table S5).

### Discussion

Experiment 1 found no evidence that participants discovered the pattern underlying the structure of the novel lexicon: Despite receiving accuracy feedback, they did not learn to use ABF in their lexical judgments. Instead, performance was guided by minBF, insofar that strings that had higher minBF were more likely to be judged as novel words. But curiously, this tendency was unlikely to have been acquired during the task, as we found no evidence that the effect of the minBF statistics increased over time (i.e., across experimental blocks). Further, the results from our additional measures also point to the possibility that participants did not engage in much local statistical learning – the individual’s capability to detect visual regularities was uncorrelated with the use of ABF or minBF (nor it was predictive of the performance in general).

One explanation for these unexpected findings is that the ABF cue was not strong enough to be detected amongst many other statistics present in our word-like stimuli. Note that ABF was manipulated in a continuous manner, meaning that some foil strings might have had very similar, or even virtually the same ABF values as words (see ***Figure 1***). Moreover, both fictitious words and foils closely resembled existing Italian words, which might have prompted participants to ignore local regularities, and instead focus on cues that are important for the processing of real words (a possibility suggested by the fact that the extent to which participants relied on the minBF cue was related to their vocabulary span). Indeed, there is some indication that the processing of novel, previously unseen letter strings may at times be driven by the statistics of one’s native language (e.g., Cassar & Treiman, 1997; Pacton, Perruchet, Fayol, & Cleeremans, 2001; Pollo, Kessler, & Treiman, 2009). Thus, we reasoned that increasing the informativity of ABF for the words vs. foils distinction could help participants learn this cue. To put this intuition to the test, and to assess the robustness of the minBF effect, we ran Experiment 2.

## Experiment 2

In Experiment 2, we once again used our lexical judgment task to investigate if readers learn the statistical patterns characterising a novel lexicon. However, we developed new stimuli to ensure that the difference in ABF between foils and word strings was more clearly pronounced (see ***Figure 1***), and tested if lexical judgments would now be affected by ABF. We also tested if the finding that readers rely on the minBF statistics would replicate with these new stimuli.

In addition, we asked if the use of the ABF and minBF statistics would be related to participants’ capability for procedural or declarative learning. A positive association with procedural learning would suggest that the cue was learnt based on the procedural memory system, and would indicate an implicit status of this cue (consistent with the hypothesis that the cue is picked up by a statistical learning mechanism), whereas an association with declarative learning would suggest that the cue was learnt based on declarative memory systems, and could indicate its explicit status in memory. To investigate these relationships, participants completed a procedural learning task (i.e., serial reaction time task; Nissen & Bullemer, 1987) and a measure of declarative learning (i.e., recognition memory task; Hedenius, Ullman, Alm, Jennische, & Persson, 2013), which have respectively been closely tied to the declarative and procedural memory systems (Janacsek et al., 2020; Reifegerste et al., 2020). Additionally, we once again measured participants’ vocabulary span and tested if it was related to their use of statistical cues.

### Methods

The stimuli, experimental scripts for the main task, raw data, and analysis scripts can be found at the project’s Open Science Framework page: *https://osf.io/vc6rw/*.

#### Participants

We recruited 42 further monolingual Italian speakers from the same population as in Experiment 1. Ethical approval and consent were arranged as per Experiment 1. Participants received 20€ on completion of the experiment.

#### Apparatus, stimuli and procedure

Experiment consisted of two sessions (1–11 days apart): In the first session, participants completed the vocabulary span measure, followed by a procedural learning task and a declarative learning task (the order of learning tasks was counter-balanced between participants; the session lasted up to 45 mins); In the second session, participants completed the lexical judgment task (90 mins). All instructions were given in Italian.

##### Mill-Hill Vocabulary Scale (MHVS)

As in Experiment 1.

##### Procedural Learning

Individual procedural learning ability was measured using a variant of Nissen and Bullemer’s (1987) serial recall task (SRT). In each trial, participants saw a visual stimulus (i.e., a yellow smiley face) appearing in one of four positions on the computer screen (i.e., left, up, right, down) and pressed the arrow key that matched the current location of the stimulus. The stimuli remained onscreen for 3000 ms or until the participant had responded (whichever occurred first), followed by a blank screen shown for 100 ms.

Stimuli were organised into 6 blocks of 60 trials. Unknown to participants, stimuli presentation varied between experimental blocks: In the first block, the position in which the stimulus appeared was determined at quasi-random (i.e., random with the constraint that position was never immediately repeated; for example, if on a given trial stimulus appeared on the left, then on the subsequent trial it could only appear in the up, right, or down position); In second to fifth blocks, stimulus presentation followed a predefined pattern similar to that used by Nissen and Bullemer (1987) (in the present study, the pattern was 1342314214, where 1 stands for left, 2 up, 3 right, and 4 down). In the sixth and final block, the stimulus presentation was again quasi-random. In the beginning of the task, participants completed 10 trials of a practice run. At the end, we included a rough check of whether participants became aware of the pattern used in the non-random blocks: They were asked to press the *y* or *n* key in response to the question “*Did you notice a pattern?*” (Italian translation: “*Hai notato delle regolarità?*”). The task was run using E-Prime (v2.0.8.90; Psychology Software Tools, Pittsburgh, PA, *www.pstnet.com*).

The typical finding from the SRT paradigm is that participants learn the presentation pattern, which leads to a decrease in response times (RTs) across the non-random blocks. Thus, following prior studies, we operationalized individual procedural learning ability as the difference between the mean RT in the last non-random block (in our task, block five) and in the final quasi-random block (block six; prior to this calculation, we trimmed RTs by removing observations exceeding 2 SD from participant mean). Higher values suggest greater sequence learning.

##### Declarative Learning

To measure declarative learning abilities, we used a recognition memory task developed by the Brain and Language Lab at Georgetown University (Hedenius et al., 2013; Lukacs, Kemény, Lum, & Ullman, 2017; Reifegerste et al., 2020). This task involved two phases. In the encoding phase, participants saw pictures of 32 real objects and 32 made-up objects presented in a quasi-random order (i.e., no more than three pictures of the same type were shown consecutively). In each trial, the computer showed a fixation cross (1000 ms), followed by a stimulus (500 ms), and participants pressed a key to indicate if the stimulus was a real or made-up object (the response window lasted for up to 5000 ms including stimulus onscreen time; the fixation cross was shown during response window; the next trial started after a response or time-out, whichever occurred first). Next, participants rested for 10 mins, after which they completed the recognition phase: Participants saw the 64 pictures from encoding intermixed with 64 new pictures (half of them showing real, half made-up objects) and indicated whether or not each stimulus was presented previously (the trial structure followed that of the encoding phase). Participants responded using the numerical keys 5 and 6. The task was programmed in E-Prime.

Declarative learning was operationalized as the d-prime sensitivity index calculated over the responses produced in the recognition phase, and computed with the standard formula d’ = z(H) – z(FA), where H is the hit rate (i.e., the number of responses where participants correctly recognized a previously presented object), and FA is the false alarm rate (i.e., the number of responses where participants incorrectly indicated recognition of an object that had not been presented before). Higher d-prime values indicate better declarative learning abilities.

##### Lexical Judgment

This task was identical to the one used in Experiment 1, with the exception that stimuli were partially replaced to ensure that foils and word strings more clearly differed from one another in terms of their ABF distributions (***Figure 1***). As in Experiment 1, the stimuli were similar to one another (i.e., foils and words were made of the same 19 letters or 150 bigrams; they were 4–6 letters long: Mdn_words_ = 6, Mdn_foils_ = 6; *U* = 19844.5, *p* = .874; they were orthographically equidistant to Italian, as measured by Coltheart’s N: Mdn_words_ = 0, Mdn_foils_ = 0; *U* = 20300, *p* = .565), and similar to real Italian words (i.e., strings were constructed from bigrams and letters taken from the Italian lexicon; letter frequencies resembled those observed in Italian; ***Table 1***).

### Statistical Analysis

To investigate if participants used the ABF pattern, and whether they relied on minBF, we re-ran the main (i.e., best-fit) GLMM from Experiment 1. The model took the form: “word” response ~ (ABF * Type * Seen) + (minBF * Type * Seen) + (ABF * Type * Seen | Participant) + (minBF * Type * Seen | Participant) + (Seen | Item). To test whether the use of ABF and minBF changed across the experimental session, we re-fit this model controlling for Block, as per Experiment 1. Prior to this analysis, we excluded 1 participant who had less than 33% observations in some design cells (leaving n = 41).

Additionally, we asked if the use of statistical cues would be related to individual differences in procedural and declarative learning ability. We tested this by re-fitting the main GLMM including the score from the procedural learning task (SRT) as a covariate and, in a separate model, re-fitting it including the score from the declarative learning task (DecLearn; covariates were scaled and centred; prior to the analysis involving declarative learning, 3 participants were excluded due to achieving scores that were low outliers in this task; leaving n = 39). The models followed the structure: “word” response ~ (SRT/DecLearn * ABF * Type * Seen) + (SRT/DecLearn * minBF * Type * Seen) + (ABF * Type * Seen | Participant) + (minBF * Type * Seen | Participant) + (Seen | Item).^1^

Finally, to test the relationship between the use of statistical cues and vocabulary span, we re-fit the main GLMM with participants’ MHVS score as a covariate, as per Experiment 1.

### Results

See ***Figure 3*** for a visual representation of the main findings and ***Table 2*** for model summaries. Despite the fact that we increased the informativity of the ABF pattern, Experiment 2 replicated the critical findings from Experiment 1: We found no significant effects involving this cue (*p*s > .189). Once again, lexical judgments were affected by minBF (i.e., the higher the minBF of a string, the greater the odds that participants judged it to be a fictitious word; *p* < .001), and the analysis found no evidence that this tendency was acquired during the experiment (i.e., there were no significant interactions involving minBF and Block; *p*s > .086). We also replicated the interaction between Type and Seen (*p* < .001), and follow-up analyses again showed that the odds of making a “word” response were higher for word strings than foils, but only when participants were judging a string they had already seen (*p* < .001) and not when judging previously unseen strings (*p* = .246; Table S2). As in Experiment 1, this implies that participants were unable to tell whether a string was a fictitious word or a foil unless they had judged it before (see Figure S1 for a detailed visualisation of this pattern).

With respect to our measures of procedural and declarative learning: Whereas declarative memory did not in any way affect the performance in the main task (*p*s > .437; full results in Table S6), there was a three-way interaction between Type, Seen and procedural learning (*p* = .012; see Table S7). Follow-up comparisons revealed that this interaction was driven by the fact that, when responding to already-seen word items, individuals with higher procedural learning scores were less likely to (correctly) judge such items as fictitious words. To put it differently, individuals with worse procedural learning were actually better at correctly identifying previously seen fictitious words as such. Procedural learning abilities had no effect on the judgments made for foils (either seen or unseen) or unseen word strings (results from the follow-up analyses in Tables S8 and S9). This unexpected pattern is difficult to interpret. But crucially, our analysis found no evidence that the individual differences in declarative or procedural learning influenced how participants used the statistical cues (i.e., there were no significant effects involving either learning faculty and ABF or minBF; *p*s > .144). Thus, overall, these results fit with our other findings – the absence of any positive effects of either declarative or procedural learning on performance is not surprising, given that there was no evidence for learning of the local statistical pattern (ABF).

Finally, our analysis of the relationship between vocabulary span (measured by MHVS) and the use of statistical cues did not replicate Experiment 1: The interaction between minBF and MHVS was now not significant (*p* = .857). Instead, there was a three-way interaction between Type, Seen, and MHVS (*p* = .020), suggesting that, whereas overall participants were more accurate when responding to seen strings (i.e., when responding to previously seen strings, participants produced a “word” response more often for word strings than foils), this effect was particularly strong for individuals with high vocabulary span, perhaps indicating their superior ability to acquire new lexical items (full results in Table S4).

### Discussion

Experiment 2 replicated the main findings of Experiment 1: Whereas there was no evidence that participants learnt the ABF pattern built into our stimuli, lexical judgments were guided by minBF. Moreover, we once again found that the tendency to use the latter cue was most likely rooted in participants’ prior knowledge: The effect of minBF on lexical judgments did not increase over time, and it was uncorrelated with individual learning abilities.

## General Discussion

In the two experiments reported here, participants saw novel letter strings and judged which strings were words in a fictitious language and which were foils. Unknown to the participants, the stimuli contained an implicit statistical cue allowing for the correct recognition of words and foils – fictitious words had higher average bigram frequency (ABF) than foils. We found no evidence that participants picked up on this cue: Consistently across experiments, there was no effect of the ABF statistic on judgments and no indication of such an effect emerging as participants progressed through the task.

However, we observed that participants based their judgments on a different statistic: The odds of judging a novel string as a word were negatively correlated with minimal bigram frequency (minBF), that is, the lower the frequency of the least frequent bigram in a string, the less likely it was that participants judged this string as a fictitious word. Crucially, given that minBF did not support correct judgments (and was not in any way reinforced during the task), this tendency appears to be rooted in participants’ knowledge of bigram probabilities in their native language. These results imply that readers rely on bigram statistics and, of course, that such statistics are coded within the word identification system in the first place.

Our findings converge with previous evidence that readers are sensitive to the regularities encoded in their native language. For example, the processing of written words is known to be facilitated by the consistency in the mapping between spelling and pronunciation (e.g., words containing letter patterns that are pronounced consistently, *l-ike* and *h-ike*, are named faster than words containing patterns that are pronounced differently in different words, *h-ave* and *g-ave*; Jared, 2002; Jared, McRae, & Seidenberg, 1990; Perry, Ziegler, & Zorzi, 2007) or in the mapping between spelling and meaning (e.g., words whose spelling provides a consistent cue to a certain meaning, *widow-er* and *widow-ed*, are processed faster compared to words with low orthography to semantics consistency, *whisk-er, whisk-y*; Marelli, Amenta, & Crepaldi, 2015).

Interestingly, a recent study suggested that sensitivity to regularities in the reading materials is directly linked to the statistical structure of one’s native language. Ulicheva et al. (2020) observed that English suffixes were consistently associated with a particular lexical category (e.g., whereas suffixes *-ous* and *-able* occur predominantly in adjectives, *-ness* and *-ment* are typical for nouns). Critically, the strength of such association had a clear impact on visual word processing: The study showed that the extent of consistency in suffix-to-category mapping predicted readers’ lexical judgments (Experiment 1), their spelling behavior (Experiment 2), and fixations while reading (Experiment 3). Based on these data, Ulicheva et al. (2020) proposed that the processing of written words is shaped by the implicit regularities that characterize language.

However, although there is sufficient evidence that readers represent the regularities between different levels of linguistic information (e.g., form to meaning, form to sound, sound to meaning), it is less clear whether they also code for the probabilities attached to linguistic units per se, in particular to lower-level units like letters and bigrams (for reviews, see Chetail, 2015; Schmalz & Mulatti, 2017).

On one hand, some authors proposed that written words are coded in terms of their constituting bigrams, and that such coding is tied to probabilistic information. For example, Dehaene et al. (2005) put forward a hierarchical coding scheme where known letter strings are represented by a network of neurons specialised in detecting particular bigrams and letters. Importantly, the acquisition of such detectors is based on co-occurrence statistics – for instance, specialised detectors should develop for the bigrams that occur particularly frequently. Similarly, Grainger and Ziegler (2011) proposed that readers identify frequently co-occurring letters and chunk them into bigram representations (amongst other, higher-level representations), which they then use to recognize words (at least when processing along the fine-grained route of orthographic processing). Moreover, there is some evidence that bigram statistics of one’s native language may impact how readers respond to nonword strings (Cassar & Treiman, 1997; Pacton et al., 2001; Pollo, Kessler, & Treiman, 2009).

But studies that investigated the role of bigram statistics in actual visual word processing produced mixed results: Whereas some reported that words made up of bigrams with low frequency are processed faster than words with high frequency bigrams (Owsowitz, 1963), other studies found such effects for high frequency bigrams (Biederman, 1966; Massaro, Jastrzembski, & Lucas, 1981) or reported null results (Andrews, 1992; Gernsbacher, 1984; Johnston, 1978; Keuleers, Lacey, Rastle, & Brysbaert, 2012; Manelis, 1974; McClelland & Johnston, 1977).

Our study contributes to this investigation by providing further evidence that readers may indeed represent the probabilistic status of bigrams, and that bigram frequencies impact the processing of strings that closely resemble real words.^2^ In this sense, our data support the claim that the word identification system utilizes statistical information (e.g., Dehaene et al., 2005; Grainger & Ziegler, 2011). Further, they are also convergent with the models suggesting that the human mind represents language in a probabilistic manner. For instance, Seidenberg (1987) proposed that sub-lexical representations (e.g., representations for morphemes) are based on the probabilistic distribution of letter patterns in the lexicon. Although this early model did not specify what type of orthographic regularity is used (e.g., occurrence frequency, transitional probability), it made a case for the importance of probabilistic information in the mental representation of language. More recently, Baayen et al. (2011) constructed a model involving a layer of simple form representations (i.e., letters and bigrams) and a layer of semantic representations (i.e., meanings), which learnt to associate forms and meanings based on the information about their co-occurrence: When form representations co-occurred with a particular meaning (e.g., *h* and *ha* with the meaning HAND), their association was strengthened (proportionally to how many other letters and bigrams were present in the input); when the form representations were present, but the particular piece of meaning was not (e.g., *h* and *ha* appearing in the word *hat*, which is unrelated to the meaning HAND), the association was weakened. Critically, the model was able to account for several effects observed in the processing of morphologically complex words (e.g., family size effect; Schreuder & Baayen, 1997), thus suggesting that the correspondence between form and meaning might be captured by the human mind in a similar manner (see also Plaut & Gonnerman, 2000; Rueckl, Mikolinski, Raveh, Miner, & Mars, 1997). Our evidence fits these models in the sense that it clearly shows that string processing can be affected by the regularities based on one’s native language.

What kind of process allows for the acquisition of linguistic regularities? The evidence for the use of probabilistic information in visual word processing has been taken to suggest that readers engage in statistical learning (Arciuli, 2018; Chetail, 2015; Christiansen, 2019; Frost, 2012; Frost et al., 2020; Ulicheva et al., 2020). More specifically, it has been proposed that readers (typically in an implicit and unsupervised fashion) learn about the regularities as they are processing the input (e.g., Christiansen & Chater, 2016). This hypothesis is motivated by the numerous studies demonstrating that even a brief exposure to visual stimuli results in robust learning of probabilistic information (e.g., Fiser & Aslin, 2001; Reber, 1989). After all, written words can be conceptualized as organized sequences of visual objects (i.e., letters or bigrams), so it is plausible that the same statistical learning mechanism might be active when processing non-linguistic and linguistic visual materials (see Lelonkiewicz et al., 2020; Siegelman, 2020).

However, in the experiments described here, we found no evidence for the learning of local regularities. Recall that there was a statistical cue built into the experimental lexicon that the participants were expected to pick up: The strings which were words in the fictitious language had a higher average bigram frequency than foil strings. Yet, we found no effect of this cue on string processing. This lack of “statistical pick-up” is by no means a novelty in the literature on implicit learning; for example, Kühnel et al. (2019) showed that participants failed to learn the relative order of color words as they were required to read them aloud. Yet, it may sound a bit surprising in the context of the literature that we discussed above; with the caveat that null evidence should be interpreted with caution, we would like to offer an explanation for this unexpected result.

We propose that participants did not in fact engage in statistical learning, because the stimuli used in our task were too similar to real words to encourage such learning. The ability to learn local statistical regularities might be particularly useful when processing stimuli that are highly unfamiliar, like strings written in an unknown script. In such circumstances, learners can ease the cognitive load by identifying the recurring patterns. But when stimuli can be linked to pre-existing mental representations (e.g., representations for familiar linguistic units in one’s native language), then the most effective way to preserve mental resources is to use this knowledge, rather than engage in on-the-fly learning.^3^

This accords with the data from the secondary tasks used in our study. First, we found a suggestion that the tendency to rely on minimal bigram frequency could be linked to linguistic knowledge: In Experiment 1, the effect that this cue had on string processing was positively related to participants’ vocabulary span (i.e., the greater the vocabulary, the stronger the effect of minimal bigram frequency; recall that minimal bigram frequency was the cue that was not reinforced by the task); that said, Experiment 2 did not replicate this finding, and so we cannot exclude the possibility that it constitutes a type I error. Second, there was no evidence that the use of statistical cues (i.e., minimal bigram frequency, average bigram frequency) in the lexical judgment task was related to declarative or procedural learning abilities, or to visual statistical learning ability. In other words, it is unlikely that participants’ tendency to rely on minimal bigram frequency could be explained by their individual capacity to learn new information, be it implicit or explicit. (However, we would like to note the fact there was no positive effect of these learning faculties does not imply that there is no role of procedural/declarative memory in reading.) Thus, these results are convergent with the possibility that regularity learning was not engaged in our experiments, because the stimuli allowed participants to rely on their pre-existing knowledge.

Under this interpretation, our data can be reconciled with the accounts proposing that statistical learning is engaged in visual word processing. Specifically, the learning of local regularities might be particularly important when processing materials that do not allow the readers to make a connection to prior linguistic knowledge. This resonates with the current evidence for the use of statistical learning in string processing: The study by Chetail (2017), which we mentioned in the Introduction, demonstrated that a brief exposure to a novel lexicon was sufficient for the participants to become sensitive to frequently recurring bigrams; Further, Lelonkiewicz et al. (2020) exposed participants to a stream of novel strings and found that they extracted the regularities determining the structure of those strings – following exposure, string processing was sensitive to the presence of chunks of frequently co-occurring characters. Critically, however, the stimuli used in these experiments were composed of entirely unfamiliar fonts (i.e., artificial script in Lelonkiewicz et al. and ancient Phoenitian alphabet in Chetail, Experiment 1), meaning that it was impossible to link them to any phonological, semantic, or syntactic representations. Thus, participants in those studies relied on the regularities in the current input.

In contrast, in the experiments reported here, the connection to linguistic knowledge was made possible (or perhaps even encouraged) by the fact that the learning lexicon resembled the native language of the participants. In consequence, string processing was guided by a cue that was external to the task and most likely came from participants’ prior experience of language use. This, again, is compatible with what is known about statistical learning. For example, Potter et al. (2017) found that participants’ ability to extract between-syllable transitional probabilities improved as a result of learning a language which puts importance on the tonal structure of words (i.e., Mandarin Chinese). Moreover, Siegelman et al. (2018) showed that statistical learning tasks which allow for a connection between the stimuli and prior knowledge (e.g., tasks involving linguistic materials) are characterized by low internal consistency, meaning that the extraction of regularities occurs at a different rate for different items used in the task (conversely, tasks where such connection cannot be made have high internal consistency). Importantly, the study also demonstrated that performance in an auditory statistical learning task was predicted by participants’ knowledge of their native language (i.e., items that were more similar to L1 were associated with better learning; Siegelman et al., Experiment 4).

In sum, it has been shown that statistical learning is modulated by the linguistic knowledge with which participants enter the task, and that such modulation is particularly likely to occur when the learning materials bear a resemblance to L1 or other known languages. Similarly, in the current study, which involved strings similar to Italian words, native speakers of Italian appeared to have prioritized the use of prior knowledge (i.e., minimal bigram frequency cue) over the learning of local regularities (i.e., average bigram frequency cue). Thus, our data suggest that the involvement of on-the-fly statistical learning in visual word processing might be limited to certain contexts. In particular, we propose that the influence of local regularities is strongest in situations when readers are faced with entirely novel materials (e.g., an Italian speaker reading Arabic script). However, when faced with materials that have some degree of familiarity (e.g., an Italian speaker reading words in an unfamiliar dialect of Italian), the processing is primarily driven by the regularities based on an already learnt language.

Finally, we turn to discussing some of the outstanding questions related to our study. First, it could be asked why was there an effect of the least frequent bigrams, rather than of high-frequency bigrams, for example (recall that our analysis found no effect of highly frequent bigrams on string processing). A possible explanation for this finding refers to a linguistic principle known as the Zipf’s law (Zipf, 1949). According to this principle (which has been subsequently confirmed by empirical and computational studies; see Perruchet, 2019; Piantadosi, 2014), a small number of highly frequent words accounts for most of the lexical tokens in language. Consequently, a small number of high frequency bigrams (i.e., bigrams that occur in highly frequent words; for instance, bigram *th* occurs in highly frequent words like *the, then, than, that*, which makes it the most frequent bigram in English) can account for most of the bigram tokens.

Notably, such distribution of bigrams and words may have important implications for visual word recognition: It should be more efficient to track infrequent rather than frequent bigrams, because it is the former that help to quickly differentiate between words (e.g., identifying the rare bigram *dk* allows for a quick recognition of the word *vodka*, whereas the frequent bigram *th* is associated with numerous words and so is less helpful with regard to word identification). It is therefore plausible that less frequent bigrams receive a special status in the visual system, and that they are used to facilitate the recognition of written words (see Westbury & Buchanan, 2002).^4^ This possibility would accord with our finding that learners relied specifically on minimal bigram frequency. However, note that our study was not designed to systematically investigate the effects of frequent vs. infrequent bigrams, and so this aspect of our findings remains peripheral to the focus of our paper (which asked whether string processing is guided by bigram frequencies at large).

One shortcoming of the present design is that it does not directly dissociate between the bigram frequencies based on the experimental stimuli and those based on participants’ knowledge of Italian words. Thus, one may wonder whether the fact that participants relied on minimal bigram frequencies can indeed be attributed to their use of pre-existing mental representations – in principle, participants could have calculated bigram frequencies based on their exposure to the novel strings. This concern seems to be dismissible given the present data: Our analyses showed that the effect of minimal bigram frequency occurred already in the earliest stages of the experimental session, arguably too early for participants to have extracted any local regularities, especially regularities that were inconsistent with the accuracy feedback provided by the task (feedback reinforced the use of the average bigram frequency cue, not minimal bigram frequency).

However, it would be very interesting to determine whether readers learn local statistics even when the connection between the current input and the pre-existing knowledge is in place. Arguably, such situations are not uncommon. For instance, consider children who, particularly in the later stages of the development, are faced with the challenge of adding new words to an already sizable vocabulary, or adults learning a new language from the same language group (e.g., Italian speakers learning French). Future research could employ a design where local statistics coincide with L1 regularities (e.g., use bigrams that are highly frequent in L1, but infrequent in experimental stimuli) and test the effects of these statistics on visual string processing.

To conclude, we found that statistical regularities may affect how readers approach new strings. However, the totality of our data brings us to suggest that statistical learning might not always engaged in visual word processing – at times, readers may rely on the pre-existing statistical knowledge of their native language, rather than on patterns specific to the stimuli at hand.

## Data Accessibility Statements

Data involved in this research can be found at: *https://osf.io/vc6rw/* DOI *10.17605/OSF.IO/VC6RW*

## Additional File

The additional file for this article can be found as follows:

10.5334/joc.209.s1 Supplementary Materials.

## Footnotes

With regard to the procedural learning task, the awareness check indicated that 32 (out of 42) participants reported noticing a pattern in the stimuli presentation. To follow-up on this finding, we re-fit the GLMM described above including data only from the participants who reported noticing a pattern. The results did not substantially differ from the overall analysis and are reported in Table S10 in the Supplement.

Although our findings show that the knowledge of bigram statistics is represented in some form, they do not imply explicit representations for bigrams (see Baayen et al., 2011). There is an entire field of literature debating what kind of representations support visual word processing. The present experiments were not designed to tackle these questions, and we do not think that our data can inform this debate in any clear direction. However, the present results imply that readers are sensitive to bigram frequency in a way that previous studies were not able to show, and models of visual word identification should account for this fact.

A similar idea is expressed in the predictive processing literature where the processing is believed to be largely based on experience-based expectations about the input, rather than the input itself (e.g., Pickering & Garrod, 2013; Dell & Chang, 2014; Wilson & Knoblich, 2005).

The predictive perspective on processing (e.g., Pickering & Garrod, 2013) provides another reason for tracking infrequent bigrams: Perceiving unexpected information is associated with a prediction error, which is then used to update the subsequent predictions made by the system. In a similar manner, seeing a rare bigram inside a word-like string could constitute a highly salient perceptual event, one that can be used to calibrate subsequent string processing (see Gagl et al., 2020).
