# Supplementary material for: Knowledge of Statistics or Statistical Learning? Readers Prioritize the Statistics of their Native Language Over the Learning of Local Regularities
Source: J Cogn. 2022 Feb 21;5(1):18. doi: 10.5334/joc.209 (PMC9400655; doi:10.5334/joc.209)
Supplement: Supplementary Materials. [file joc-5-1-209-s1.pdf]

Supplementary Materials

Contents

Investigating the Effect of Type in Seen vs. Unseen Strings 2
Investigating the Effect of Vocabulary Span 4
Investigating the Effect of VSL 6
Investigating the Effect of Declarative Learning 8
Investigating the Effect of Procedural Learning 9
Model Controlling for String Length 13
Model Controlling for Orthographic Similarity (Coltheart´s N) 15
Model Including ABF and Model Including ABF and maxBF 17
Pilot Studies 19
Participants, Methods and Results 19

Investigating the Effect of Type in Seen vs. Unseen Strings

As reported in the main text, we followed-up on the Type * Seen interaction observed in Experiments 1-2: We wanted to determine if participants' ability to distinguish between fictitious words and foils differed for strings that had been previously seen vs. those that had not. To this end, we re-fit our main model (i.e., the one including ABF and minBF) separately for seen and unseen strings (as a consequence, we no longer included Seen as a predictor in the model). We found that, for either experiment, the effect of Type was significant only in seen strings (see Tables S1 and S2 below). Figure S1 below illustrates how the ability to distinguish between fictitious words and foils changed between the first, second, and third presentation of each item. Although the numerical pattern suggests that participants produced somewhat more 'word' responses to fictitious words than foils already upon the first presentation, there is considerable variability – a likely explanation of why our statistical analyses did not confirm this pattern.

Table S1. Experiment 1: Results from follow-up models investigating the effect of Type for seen vs. unseen strings.

	Experiment 1							
	seen strings				unseen strings			
	$\hat{\beta}$	*SE*	*z*	*p*	$\hat{\beta}$	*SE*	*z*	*p*
Intercept	.56	.08	6.80	< **.001**	.02	.10	.25	.806
ABF	.01	.05	.31	.759	.06	.05	1.20	.228
Type	-.83	.10	-8.29	< **.001**	.03	.07	.45	.651
minBF	.04	.02	1.94	.052	.06	.03	2.32	**.020**
ABF * Type	.12	.06	1.80	.072	.00	.07	-.05	.958

minBF * Type	.06	.05	1.16	.244	.16	.06	2.40	**.016**

Table S2. Experiment 2: Results from follow-up models investigating the effect of Type for seen vs. unseen strings.

	Experiment 2							
	seen strings				unseen strings			
	$\hat{\beta}$	*SE*	*z*	*p*	$\hat{\beta}$	*SE*	*z*	*p*
Intercept	.41	.08	5.05	< **.001**	-.04	.08	-.49	.621
ABF	.21	.05	3.98	< **.001**	.10	.06	1.60	.110
Type	-.61	.12	-5.02	< **.001**	.10	.09	1.16	.246
minBF	.03	.04	1.13	.258	.10	.03	3.60	< **.001**
ABF * Type	-.15	.08	-1.75	.080	-.02	.09	-.25	.799
minBF * Type	.07	.04	1.59	.113	.04	.05	.95	.340

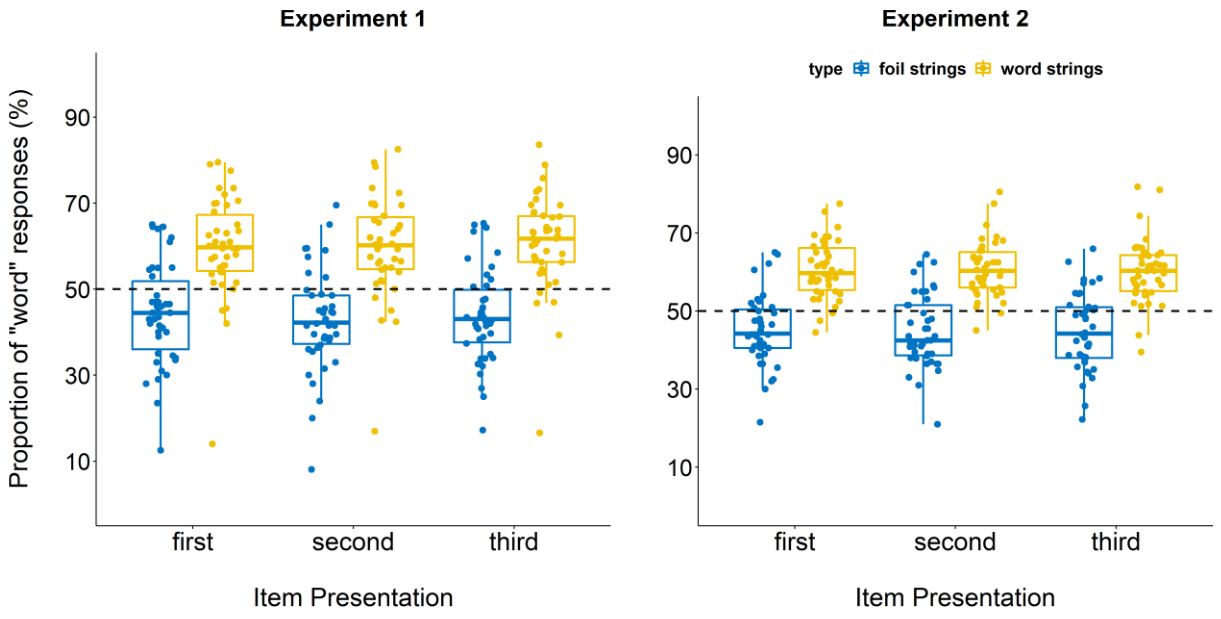

Figure S1. Experiments 1-2: Mean percentages of “word” responses for each participant, shown by Type (foil vs. word strings) and Item Presentation (first vs. second vs. third). The dashed line mark 50%

Investigating the Effect of Vocabulary Span

Table S3. Experiment 1: Results from the fixed effects structure of the GLMM including MHVS (vocabulary span), ABF, minBF, Type (foil vs. word) and Seen (unseen vs. seen).

Experiment 1				
	$\hat{\beta}$	*SE*	*z*	*p*
Intercept	.04	.08	.47	.636
MHVS	.07	.09	.72	. 474
ABF	.05	.06	.92	. 355
Type	-.04	.07	-.65	. 516
Seen	-.31	.07	-4.07	**< .001**
minBF	.18	.05	3.72	**< .001**
MHVS * ABF	.01	.07	.12	.902
MHVS * Type	.03	.08	.35	.724
ABF * Type	.02	.07	.34	.730
MHVS * Seen	-.11	.08	-1.49	.137
ABF * Seen	.08	.07	1.03	.301
Type * Seen	.86	.12	7.30	**< .001**
minBF * MHVS	.15	.06	2.67	**.008**
minBF * Type	-.12	.05	-2.27	**.023**

minBF * Seen	-.10	.06	-1.53	.127
MHVS * ABF * Type	-.06	.08	-.76	.450
MHVS * ABF * Seen	-.03	.07	-.37	.709
MHVS * Type * Seen	.17	.12	1.39	.164
ABF * Type * Seen	-.13	.09	-1.40	.160
minBF * Type * MHVS	-.12	.06	-1.95	.051
minBF * Seen * MHVS	-.03	.06	-.45	.652
minBF * Type * Seen	.08	.07	1.13	.258
MHVS * ABF * Type * Seen	.08	.09	.85	.396
MHVS * minBF * Type * Seen	.00	.07	-.05	.956

Table S4. Experiment 2: Results from the fixed effects structure of the GLMM including MHVS (vocabulary span), ABF, minBF, Type (foil vs. word) and Seen (unseen vs. seen).

Experiment 2				
	$\beta^{\wedge}$	*SE*	*z*	*p*
Intercept	.06	.08	.76	.447
MHVS	.00	.08	-.02	.983
ABF	.10	.06	1.62	.106
Type	-.13	.07	-1.71	.087
Seen	-.30	.09	-3.10	**.002**
minBF	.14	.04	4.00	< **.001**
MHVS * ABF	.09	.06	1.43	.152
MHVS * Type	-.11	.08	-1.34	.180

ABF * Type	.02	.08	.28	.779
MHVS * Seen	-.12	.09	-1.37	.171
ABF * Seen	-.03	.09	- .36	.718
Type * Seen	.78	.14	5.71	< **.001**
minBF * MHVS	-.01	.04	-.18	.857
minBF * Type	-.04	.04	-0.93	.353
minBF * Seen	-.05	.05	-.100	.315
MHVS * ABF * Type	.00	.09	.02	.987
MHVS * ABF * Seen	-.05	.08	-.65	.518
MHVS * Type * Seen	.31	.13	2.33	**.020**
ABF * Type * Seen	.14	.11	1.22	.221
minBF * Type * MHVS	.03	.04	.83	.404
minBF * Seen * MHVS	.00	.04	-.01	.994
minBF * Type * Seen	-.03	.06	-.51	.613
MHVS * ABF * Type * Seen	-.04	.10	-.39	.697
MHVS * minBF * Type * Seen	-.03	.05	-.54	.586

Investigating the Effect of VSL

Table S5. Experiment 1: Results from the fixed effects structure of the GLMM including VSL (visual statistical learning), ABF, minBF, Type (foil vs. word) and Seen (unseen vs. seen).

	Experiment 1			
	$\hat{\beta}$	*SE*	*z*	*p*

Intercept	.03	.08	.43	.664
VSL	-.09	.08	-1.21	.228
ABF	.06	.06	1.12	.262
Type	-.04	.06	-.63	.529
Seen	-.31	.08	-4.20	< **.001**
minBF	.20	.05	3.98	< **.001**
VSL * ABF	.02	.06	.42	.673
VSL * Type	.05	.06	.82	.415
ABF * Type	.00	.07	.04	.968
VSL * Seen	.01	.07	.13	.900
ABF * Seen	.07	.07	1.03	.303
Type * Seen	.88	.12	7.40	< **.001**
minBF * VSL	-.03	.05	-.64	.519
minBF * Type	-.15	.06	-2.59	**.009**
minBF * Seen	-.10	.06	-1.63	.103
VSL * ABF * Type	-.01	.07	-.15	.877
VSL * ABF * Seen	.00	.06	-.04	.966
VSL * Type * Seen	.10	.11	.91	.363
ABF * Type * Seen	-.12	.09	-1.33	.185
minBF * Type * VSL	.03	.06	.53	.594
minBF * Seen * VSL	.07	.05	1.21	.226
minBF * Type * Seen	.09	.07	1.27	.203
VSL * ABF * Type * Seen	.02	.08	.29	.772

VSL * minBF * Type * Seen	-.08	.06	-1.26	. 209

Investigating the Effect of Declarative Learning

Table S6. Experiment 2: Results from the fixed effects structure of the GLMM including D (declarative learning), ABF, minBF, Type (foil vs. word) and Seen (unseen vs. seen).

	Experiment 2			
	$\hat{\beta}$	*SE*	*z*	*p*
Intercept	.07	.09	.80	.421
D	-.02	.08	-.37	.714
ABF	.09	.08	1.16	.247
Type	-.12	.09	-1.22	.221
Seen	-.29	.09	-3.28	**.001**
minBF	.16	.04	3.67	**< .001**
D * ABF	.02	.06	.35	.730
D * Type	.04	.08	.52	.600
ABF * Type	.03	.10	.31	.755
D * Seen	.02	.09	.27	.788
ABF * Seen	-.03	.08	-.34	.733
Type * Seen	.77	.13	5.80	**< .001**
minBF * D	.01	.03	.36	.718
minBF * Type	-.05	.05	-.97	.331
minBF * Seen	-.05	.04	-1.16	.247
D * ABF * Type	.02	.08	.27	.783

D * ABF * Seen	.00	.08	-.02	.986
D * Type * Seen	.01	.13	.11	.910
ABF * Type * Seen	.12	.10	1.26	.209
minBF * Type * D	-.02	.04	-.54	.592
minBF * Seen * D	-.01	.04	-.23	.816
minBF * Type * Seen	-.02	.05	-.47	.635
D * ABF * Type * Seen	.01	.10	.15	.879
D * minBF * Type * Seen	.04	.05	.78	.438

Investigating the Effect of Procedural Learning

To test whether the use of statistical cues in our task was related to the ability to learn information procedurally, we re-fit our main GLMM including also the measure of procedural learning. This analysis, discussed in the main text of our paper and reported in details in Table S7 below, revealed a three-way interaction between P (procedural learning), Type and Seen. To further explore this interaction, we ran the follow-up models reported in Tables S8 and S9. All in all, we found that procedural learning impacted the processing of previously seen word strings. See the main text for an interpretation of these findings.

In addition, we re-ran the GLMM investigating procedural learning this time including data only from participants who reported noticing a pattern in the procedural learning task. The results, reported in Table S10, do not differ substantially from the analysis on the full sample: The model replicates the effect of minBF and the Type * Seen interaction, and the three-way interaction P * Type * Seen is still noticeable (although here it does not reach the conventional significance threshold, possibly because using a subset of data resulted in a lower statistical power).

Table S7. Experiment 2: Results from the fixed effects structure of the GLMM including P (procedural learning), ABF, minBF, Type (foil vs. word) and Seen (unseen vs. seen), conducted on data from full participant sample.

	Experiment 2			
	β^	*SE*	*z*	*p*
Intercept	.06	.07	.87	.384
P	-.11	.07	-1.58	.113
ABF	.08	.06	1.36	.175
Type	-.10	.07	-1.41	.159
Seen	-.26	.09	-2.79	**.005**
minBF	.14	.03	4.13	< **.001**
P * ABF	-.08	.06	-1.46	.145
P * Type	.09	.07	1.16	.247
ABF * Type	.02	.08	.32	.751
P * Seen	.17	.08	2.03	**.042**
ABF * Seen	-.01	.09	-.13	.896
Type * Seen	.71	.13	5.33	< **.001**
minBF * P	.00	.03	-.15	.877
minBF * Type	-.04	.04	-1.15	.248
minBF * Seen	-.05	.05	-1.00	.319
P * ABF * Type	.07	.08	.88	.377
P * ABF * Seen	.08	.07	1.12	.264
P * Type * Seen	-30	.12	-2.50	**.012**

ABF * Type * Seen	.12	.12	1.13	.256
minBF * Type * P	.01	.04	.16	.869
minBF * Seen * P	-.03	.04	-.71	.479
minBF * Type * Seen	-.03	.05	-.46	.648
P * ABF * Type * Seen	-.07	.10	-.74	.459
P * minBF * Type * Seen	.02	.05	.43	.669

Table S8. Experiment 2: Results from follow-up models investigating the effect of P (procedural learning) for seen vs. unseen strings.

	Experiment 2							
	seen strings				unseen strings			
	$\hat{\beta}$	*SE*	*z*	*p*	$\hat{\beta}$	*SE*	*z*	*p*
Intercept	-.20	.08	-2.33	**.020**	.06	.09	.73	.466
P	.06	.08	.77	.441	-.12	.08	-1.52	.123
ABF	.07	.06	1.03	.305	.08	.07	1.10	.270
Type	.61	.11	5.28	< **.001**	-.10	.09	-1.16	.246
minBF	.10	.04	2.57	**.010**	.14	.04	3.58	< **.001**
P * ABF	.00	.05	-.02	.983	-.08	.06	-1.46	.143
P * Type	-.22	.10	-2.16	**.031**	.08	.07	1.17	.243
ABF * Type	.15	.08	1.75	.079	.02	.09	.25	.800
minBF * P	-.04	.03	-1.21	.225	.00	.03	-.15	.877
minBF * Type	-.07	.04	-1.60	.110	-.04	.05	-.96	.338
P * ABF * Type	.00	.06	-.03	.975	.07	.08	.92	.359

P* minBF * Type	.03	.03	.83	.408	.01	.04	.18	.855

Table S9. Experiment 2: Results from follow-up models investigating the effect of P (procedural learning) for previously seen word strings vs. previously seen foils.

	Experiment 2							
	seen word strings				seen foil strings			
	β^	*SE*	*z*	*p*	*β*^	*SE*	*z*	*p*
Intercept	.41	.07	5.48	< **.001**	-.20	.09	-2.31	**.021**
P	-.16	.06	-2.50	**.012**	.06	.08	.76	.446
ABF	.22	.05	4.01	< **.001**	.07	.06	1.02	.308
minBF	.02	.02	1.07	.285	.10	.04	2.56	**.010**
P * ABF	.00	.04	-.02	.984	.00	.05	-.02	.983
P * minBF	.00	.02	-.33	.741	-.04	.03	-1.22	.222

Table S10. Experiment 2: Results from the fixed effects structure of the GLMM including P (procedural learning), ABF, minBF, Type (foil vs. word) and Seen (unseen vs. seen), conducted on data from participants who reported noticing a pattern in the procedural learning task.

	Experiment 2			
	β^	*SE*	*z*	*p*
Intercept	.06	.10	.65	.517
P	-.13	.09	-1.50	.133

ABF	.05	.09	.59	.557
Type	-.09	.11	-.90	.370
Seen	-.23	.09	-.250	**.012**
minBF	.13	.05	2.57	**.010**
P * ABF	-.06	.07	-.85	.391
P * Type	.11	.09	1.23	.218
ABF * Type	.05	.11	.44	.661
P * Seen	.10	.09	1.10	.270
ABF * Seen	.00	.08	-.02	.982
Type * Seen	.65	.13	5.02	< **.001**
minBF * P	-.01	.04	-.27	.785
minBF * Type	-.02	.06	-.35	.728
minBF * Seen	-.01	.05	-.31	.754
P * ABF * Type	.00	.09	.05	.961
P * ABF * Seen	.02	.08	.22	.827
P * Type * Seen	-.19	.13	-1.50	.133
ABF * Type * Seen	.13	.11	1.20	.230
minBF * Type * P	.00	.04	-.07	.947
minBF * Seen * P	-.03	.05	-.62	.536
minBF * Type * Seen	-.08	.06	-1.35	.175
P * ABF * Type * Seen	.00	.11	.04	.971
P * minBF * Type * Seen	.03	.05	.62	.534

Model Controlling for String Length

Given that word length is known to affect visual word processing (e.g., New, Ferrand, Pallier, & Brysbaert, 2006), we wanted to ensure that the findings from our main analyses were not confounded by the fact that the strings varied in length. To test this, we re-fit our main model but this time we also included Length (i.e., string length counted in letters) as an additional predictor. For either Experiment 1 and 2, these analyses replicated our main findings, thus confirming that length was not a confounding factor (see Tables S11 and S12 below).

Table S11. Experiment 1: Results from the fixed effects structure of the GLMM controlling for string length.

	Experiment 1			
	$\hat{\beta}$	*SE*	*z*	*p*
Intercept	-.21	.21	-.97	.333
Length	.26	.19	1.38	.166
ABF	.05	.06	.96	.339
Type	-.03	.06	-.51	.607
Seen	-.32	.07	-4.30	**< .001**
minBF	.21	.05	4.08	**< .001**
ABF * Type	.01	.06	.12	.906
ABF * Seen	.07	.07	.99	.324
Type * Seen	.87	.12	7.38	**< .001**
minBF * Type	-.15	.06	-2.66	**.008**
minBF * Seen	-.11	.06	-1.75	.079

ABF * Type * Seen	-.11	.09	-1.27	.202
minBF * Type * Seen	.09	.07	1.31	.190

Table S12. Experiment 2: Results from the fixed effects structure of the GLMM controlling for string length.

	Experiment 2			
	$\hat{\beta}$	*SE*	*z*	*p*
Intercept	.36	.22	1.62	.105
Length	-.30	.20	-1.51	.131
ABF	.07	.07	1.06	.290
Type	-.10	.09	-1.15	.248
Seen	-.26	.09	-2.96	**.003**
minBF	.14	.04	3.45	**< .001**
ABF * Type	.02	.10	.25	.803
ABF * Seen	-.01	.07	-.14	.887
Type * Seen	.70	.13	5.50	**< .001**
minBF * Type	-.04	.05	-.85	.396
minBF * Seen	-.05	.04	-1.15	.251
ABF * Type * Seen	.12	.10	1.31	.190
minBF * Type * Seen	-.02	.05	-.51	.612

Model Controlling for Orthographic Similarity (Coltheart´s N)

To ensure that our findings were not confounded by orthographic similarity (between our stimuli and the Italian lexicon), we refit our main statistical model controlling for Coltheart´s N. We found no effect of this measure on lexical judgments and the main pattern of results remained unchanged (i.e., the effect of minBF was once again statistically significant; the effect of ABF was not). See Tables S13 and S14 below.

Table S13. Experiment 1: Results from the fixed effects structure of the GLMM controlling for orthographic similarity (Coltheart´s N).

	Experiment 1			
	$\hat{\beta}$	*SE*	*z*	*p*
Intercept	.06	.08	.76	.448
Coltheart´s N	-.03	.02	-1.30	.192
ABF	.05	.06	1.06	.290
Type	-.04	.06	-.58	.559
Seen	-.32	.07	-4.33	< **.001**
minBF	.21	.05	4.12	< **.001**
ABF * Type	.01	.06	.04	.970
ABF * Seen	.07	.07	.98	.326
Type * Seen	.87	.12	7.40	< **.001**
minBF * Type	-.15	.06	-2.70	**.007**
minBF * Seen	-.11	.06	-1.76	.079
ABF * Type * Seen	-.11	.09	-1.27	.202
minBF * Type * Seen	.09	.07	1.31	.190

Table S14. Experiment 2: Results from the fixed effects structure of the GLMM controlling for orthographic similarity (Coltheart´s N).

	Experiment 2			
	$\hat{\beta}$	*SE*	*z*	*p*
Intercept	.06	.08	.80	.426
Coltheart´s N	.03	.03	.88	.378
ABF	.08	.06	1.32	.187
Type	-.11	.07	-1.46	.145
Seen	-.26	.09	-2.78	**.005**
minBF	.14	.03	4.11	< **.001**
ABF * Type	.02	.08	.31	.753
ABF * Seen	-.01	.08	-.13	.897
Type * Seen	.71	.14	5.12	< **.001**
minBF * Type	-.04	.04	-1.10	.271
minBF * Seen	-.05	.05	-1.01	.314
ABF * Type * Seen	.12	.11	1.15	.251
minBF * Type * Seen	-.02	.06	-.45	.652

Model Including ABF and Model Including ABF and maxBF

As discussed in the main text, prior to establishing that our data are best represented by the GLMM including both ABF and minBF (i.e., our main model reported in this paper provided the best fit), we ran two other models: A GLMM including ABF, and a GLMM including ABF and maxBF. The results from these models are reported in Tables S15 and S16 below.

Table S15. Experiment 1: Results from the fixed effects structure of the GLMM including ABF, Type (foil vs. word) and Seen (unseen vs. seen).

Experiment 1				
	$\hat{\beta}$	*SE*	*z*	*p*
Intercept	.01	.08	.11	.914
ABF	.11	.06	1.89	.058
Type	.00	.06	.00	.999
Seen	-.30	.07	-3.99	< **.001**
ABF * Type	.00	.06	-.01	.992
ABF * Seen	.04	.07	.65	.515
Type * Seen	.84	.12	7.20	< **.001**
ABF * Type * Seen	-.10	.08	-1.18	.236

Table S16. Experiment 1: Results from the fixed effects structure of the GLMM including ABF, maxBF, Type (foil vs. word) and Seen (unseen vs. seen).

Experiment 1				
	$\hat{\beta}$	*SE*	*z*	*p*
Intercept	-.01	.07	-.19	.852
ABF	.06	.06	1.01	.311
Type	.02	.07	.30	.762
Seen	-.28	.08	-3.64	< **.001**
maxBF	.04	.03	1.32	.187

ABF * Type	.04	.07	.61	.540
ABF * Seen	.07	.08	.81	.415
Type * Seen	.83	.12	6.88	**< .001**
maxBF * Type	-.03	.04	-.91	.362
maxBF * Seen	-.02	.04	-.57	.567
ABF * Type * Seen	-.13	.10	-1.38	.167
maxBF * Type * Seen	.06	.05	1.12	.261

Pilot Studies

Participants, Methods and Results

To validate our procedure and materials, we conducted two miniature pilot studies. First, we recruited 4 further participants who took part in a version of our lexical judgment task where foil strings were never repeated, and word strings were repeated 10 times each. A descriptive analysis of data suggested that this design primed participants to follow a simple strategy: They responded ‘word’ for any string they had seen, and responded ‘foil’ to any string they had not previously seen. We thus decided to modify the procedure and tested further 3 participants on a task which was identical to Experiment 1 (i.e., both words and foils were repeated 3 times each). A descriptive analysis of the performance did not suggest any obvious flaws of this design, and so it was used in Experiments 1-2.

References

New, B., Ferrand, L., Pallier, C., & Brysbaert, M. (2006). Re-examining the word length effect in visual word recognition: New evidence from the English Lexicon Project. *Psychonomic Bulletin and Review*, *13*(1), 45-52.
